# Evaluation of Injury Severity and Resource Utilization in Pediatric Firearm and Sharp Force Injuries

**DOI:** 10.1001/jamanetworkopen.2019.12850

**Published:** 2019-10-09

**Authors:** Ashley E. Wolf, Michelle M. Garrison, Brianna Mills, Titus Chan, Ali Rowhani-Rahbar

**Affiliations:** 1 Division of Critical Care Medicine, Seattle Children’s Hospital, University of Washington, Seattle; 2 School of Public Health, Department of Health Services, University of Washington, Seattle; 3 Seattle Children’s Research Institute, Seattle, Washington; 4 Firearm Injury and Policy Research Program, Harborview Injury Prevention and Research Center, University of Washington, Seattle; 5 School of Public Health, Department of Epidemiology, University of Washington, Seattle

## Abstract

**Importance:**

Pediatric firearm injuries are a serious and growing public health problem, constituting the second leading cause of death in children and adolescents in the United States. Firearm injuries have a high case fatality, but knowledge is limited to date regarding their injury severity and health care utilization burden compared with those of other penetrating injuries, especially among children with critical injury.

**Objective:**

To describe and compare the resource utilization, injury severity, and short-term clinical outcomes associated with pediatric firearm injuries and other penetrating trauma.

**Design, Setting, and Participants:**

This retrospective cohort study used data from the National Trauma Data Bank, an encounter-level registry of trauma data in the United States, from January 1, 2007, to December 31, 2016. Encounters for firearm injury (n = 25 155) or cut or pierce injury (21 270) in children 17 years or younger were analyzed. Statistical analysis was conducted from July 15, 2018, to June 5, 2019.

**Exposures:**

Firearm injury compared with cut or pierce injury encounters.

**Main Outcomes and Measures:**

Intensive care unit (ICU) admission, hospital and ICU length of stay (LOS), and Injury Severity Score (ISS).

**Results:**

A total of 25 155 firearm injury encounters and 21 270 cut or pierce injury encounters were analyzed. Most firearm and cut or pierce injuries occurred among boys (21 573 [85.8%] and 15 864 [74.6%]) and adolescents aged 15 to 17 years (18 807 [74.8%] and 10 895 [51.2%]). A greater proportion of those with firearm injuries were African American children compared with those with cut or pierce injuries (15 019 [61.3%] vs 6397 [31.2%]). A greater proportion of those with firearm injuries compared with cut or pierce injuries were admitted to the ICU (7682 [30.5%] vs 2712 [12.8%]). Compared with cut or pierce injuries, firearm injuries were associated with a higher mean (SD) ISS (4.6 [6.8] vs 10.9 [12.7] points), longer mean (SD) hospital LOS (2.8 [4.1] vs 5.0 [8.4] days), and longer mean (SD) ICU LOS (3.1 [4.5] vs 5.1 [7.7] days). Firearm injuries accounted for 126 027 hospital days and 39 255 ICU days, whereas cut or pierce injuries accounted for 58 705 hospital days and 8353 ICU days. After adjustments for age, sex, year, and hospital, those with firearm injuries were more likely to require ICU admission (relative risk [RR], 2.3; 95% CI, 2.1-2.5; *P* < .001) and to have higher ISS scores (6.7 points higher for all injuries; 95% CI, 6.1-7.2) compared with those with cut or pierce injuries, even among critical injuries. Multinomial logistic regression demonstrated higher risk of prolonged hospital LOS (RR ratio, 4.11; 95% CI, 3.46-4.89; *P* < .001) and ICU LOS (RR ratio, 2.2; 95% CI, 1.9-2.3) for firearm injuries compared with cut or pierce injuries.

**Conclusions and Relevance:**

This study found that pediatric firearm injuries were associated with greater severity and health care utilization compared with penetrating trauma from other mechanisms, suggesting that the mechanism of injury is an important consideration in penetrating sharp force trauma in children and adolescents. Public health measures, legislative efforts, and safe storage practices are among the interventions needed to reduce pediatric firearm injuries.

## Key Points

**Question:**

Do injury severity and resource utilization differ between firearm injuries and other penetrating trauma mechanisms in children in the United States?

**Findings:**

In this cohort study of 25 155 firearm and 21 270 cut or pierce encounters in US children 17 years or younger, critical and noncritical firearm injuries were associated with higher likelihood of intensive care unit admission, a higher Injury Severity Score, and higher likelihood of longer hospital or intensive care unit length of stay compared with cut or pierce injuries.

**Meaning:**

This study suggests greater injury severity and resource utilization associated with pediatric firearm injuries when compared with other penetrating trauma, even among children with critical injuries.

## Teaser

This cohort study compares the resource utilization, clinical outcomes, and severity of injuries from a firearm vs other penetrating mechanisms among children and adolescents in the United States.

## Introduction

Firearm injuries and deaths constitute a serious public health problem in the United States. In children and adolescents up to 19 years of age, firearm-related injuries are the second leading cause of death, accounting for 3155 deaths and 17 223 nonfatal injuries in 2016 and 3443 deaths and 18 227 nonfatal injuries in 2017.^1,2,3^ Unintentional injury deaths, homicide rates, and suicide rates from firearms among adolescents have increased substantially in recent years, with higher death rates in all 3 areas and in total firearm injuries occurring from 2013 to 2016.^4,5,6,7,8,9,10,11,12^ Pediatric firearm injuries and deaths have disproportionate implications for certain populations. The burden of violent assault deaths and injuries from firearms is largely on males, African American and Hispanic people, and socioeconomically disadvantaged persons and communities. Conversely, the burden of self-inflicted firearm suicide is higher among white children.^1,5,11,12,13,14,15,16,17,18,19,20,21,22^

Despite the growing knowledge of the epidemiologic and sociodemographic factors associated with pediatric firearm mortality, less attention has been focused on children and adolescents who sustain firearm injuries that are not initially fatal at the scene of encounter, especially those who are critically injured from firearm injuries. This population potentially represents a large burden of health care utilization and morbidity.^23,24^ Previous studies have focused on clinical outcomes in the context of mortality or have compared outcomes on the basis of intent or race/ethnicity.^13,16,17,19,25,26,27^ In addition, US society’s understanding of the potentially distinct medical consequences and morbidity of firearm injuries owing to their typically severe nature is limited to date. Previous studies have documented the high case fatality associated with firearm injuries; however, a robust comparison of indicators of severity between firearm injuries and other penetrating trauma mechanisms among children and adolescents is needed to capture a complete picture of the consequences of firearm injuries.^28,29^

In this study, we sought to determine and quantify the resource utilization, short-term clinical outcomes, and injury severity in pediatric firearm injuries that required medical care through examining nonfatal firearm encounters and injuries that survived to hospital presentation but were ultimately fatal. In particular, we aimed to enhance the understanding about firearm encounters that resulted in critical injury. We compared these injuries with other nonfirearm penetrating sharp force injuries to contextualize the outcomes for firearm encounters.

## Methods

We conducted a retrospective cohort study using the National Trauma Data Bank (NTDB) for the study period of January 1, 2007, through December 31, 2016. The NTDB is an encounter-level trauma registry with voluntary participation that was developed by the American College of Surgeons in 1989 and now represents the largest collection of trauma data in the United States. In 2016, 780 hospitals contributed pediatric data; 36 of these hospitals were pediatric-only facilities, and 306 had an association with a pediatric hospital. Although not comprehensive, the NTDB captures most of the pediatric trauma encounters in the United States. The period for analysis in this study represents the most recently available 10 years of data from the NTDB at the time of our statistical analysis from July 15, 2018, to June 5, 2019. This study was deemed exempt from review by the University of Washington Institutional Review Board because all of the data used were deidentified. No informed consent was obtained for this reason. We followed the Strengthening the Reporting of Observational Studies in Epidemiology (STROBE) reporting guidelines.^30^

Injury encounter identification was performed through the use of external causes of injury codes (E codes; a component of *International Classification of Diseases* coding). The cohort was restricted to encounters with E codes for primary injury mechanism of *firearm* as the primary population of interest and *cut/pierce* as the comparison population. Encounters with both firearm and cut or pierce E codes were excluded from analysis. The cohort was restricted to encounters for patients younger than 18 years. Encounters that were coded as *transferred* were also excluded to eliminate the risk of double counting in deidentified encounter-level data. Encounters that required admission to the hospital and encounters that were treated in the emergency department and then discharged were included.

The variables accessed for each encounter included age, race/ethnicity, sex, and insurance status, which were collected and recorded by a trauma registrar at each hospital, in addition to injury intent mechanism. We chose to assess race/ethnicity in this study to allow for a comparison with published pediatric firearm studies^9,12,13,15,17^ that demonstrated substantial differences in injury pattern and frequency by race/ethnicity. We also collected data on intensive care unit (ICU) admission, which was defined as any length of stay (LOS) in the ICU; hospital and ICU LOS; ventilator use; and Injury Severity Score (ISS). The ISS is an anatomic scoring system used in trauma patients to estimate and risk-stratify morbidity and mortality. The ISS ranges from 0 to 75 points, with injuries scored lower than 9 considered to be minor, 9 to 15 considered moderate, higher than 15 considered severe, and 75 deemed unsurvivable.^31,32^

### Statistical Analysis

Descriptive analyses were performed to obtain mean, frequency, and percentage for demographic and clinical characteristics for all injuries and critical injuries (critical was defined as injuries resulting in ICU admission). Age categories were chosen in accordance with the Centers for Disease Control and Prevention pediatric age categories: 0 to 4 years, 5 to 9 years, 10 to 14 years, and 15 to 17 years.

Injury encounters for firearm injury mechanisms were compared with injury encounters for cut or pierce injury mechanisms using regression models controlling for age, sex, year, and clustering by hospital for primary outcome variables of ICU admission, hospital and ICU LOS, and ISS. All regression analyses were 2-sided with a statistical significance level set at *P* = .05. A binomial regression model was used to compare the probability of ICU admission between the 2 groups. A generalized linear regression model with γ family and log link was used to compare the ISS between the 2 groups. A multinomial logistic regression model was used to compare the risk for distinct categories of hospital LOS and ICU LOS between the 2 groups, including relative risk (RR) ratios and their corresponding 95% CIs.

We performed a multinomial logistic regression for hospital LOS and ICU LOS because we believed that hospital LOS and ICU LOS comprised distinct subcategories reflecting different injury outcomes whose association with the injury mechanism was not ordered. As a result, we hypothesized that the association between injury mechanism and LOS would be best described by a nonlinear, nonordinal function that would yield a more comprehensive understanding of the underlying association. Distinct categories of LOS (1 day, 2-3 days, 4-9 days, and ≥10 days for hospital; 1 day, 2-3 days, and ≥4 days for ICU) were chosen after consideration of clinical relevance and to ensure adequate sample size within each category and subcategory of hospital LOS and ICU LOS by age category, allowing for model convergence.

We conducted sensitivity analyses to evaluate the stability of the associations observed, after restricting the sample in 2 ways and performing an additional analysis for LOS. First, we restricted the sample to exclude injury encounters with an ISS of 75 points, a score deemed unsurvivable and which would capture those patients likely to die of their injuries and potentially represent a large burden of injury severity. Second, we restricted the sample to exclude injury encounters whose mechanism was coded as self-inflicted, given that many self-inflicted firearm injuries are suicide attempts in the form of close-range shots to the head and face. We hypothesized that these self-inflicted injuries may represent a high injury severity and mortality risk among patients who survived to hospital admission. To evaluate the stability of the association observed for multinomial logistic regression modeling of hospital LOS and ICU LOS, we conducted a sensitivity analysis using Poisson regression with robust SEs. All statistical analyses were performed with Stata/SE, version 14.2 (StataCorp LLC), and graphs were generated with Excel, version 16.24 (Microsoft Corp).

## Results

Over the 10-year study period, we identified 25 155 encounters for firearm injuries and 21 270 encounters for cut or pierce injuries. A greater proportion of those with firearm injuries compared with those with cut or pierce injuries were male (21 573 [85.8%] vs 15 864 [74.6%]). Most firearm injuries and cut or pierce injuries were sustained by adolescents aged 15 to 17 years (18 807 [74.8%] and 10 895 [51.2%]). A greater proportion of those with cut or pierce injuries compared with those with firearm injuries were children aged 0 to 4 years (2912 [13.7%] vs 1144 [4.6%]), 5 to 9 years (2888 [13.6%] vs 1028 [4.1%]), and 10 to 14 years (4575 [21.5%] vs 4176 [16.6%]). A greater proportion of those with firearm injuries were African American children compared with those with cut or pierce injuries (15 019 [61.3%] vs 6397 [31.1%]). A greater proportion of firearm injuries were the result of assault compared with cut or pierce injuries (19 057 [79.9%] vs 8543 [40.6%]). Most cut or pierce injuries were unintentional compared with firearm injuries (10 629 [50.5%] vs 3606 [15.1%]) (Table 1).

**Table 1.** Demographic Characteristics of Patients for All and Critical Injuries

Variable	Frequency, No. (%)					
	All Injuries			Critical Injuries		
	Firearm (n = 25 155)	Cut or Pierce (n = 21 270)	Total (N = 46 425)	Firearm (n = 7682)	Cut or Pierce (n = 2712)	Total (n = 10 394)
Age, y						
0-4	1144 (4.6)	2912 (13.7)	4056 (8.7)	373 (4.9)	298 (11.0)	671 (6.5)
5-9	1028 (4.1)	2888 (13.6)	3916 (8.5)	299 (3.9)	217 (8.0)	516 (5.0)
10-14	4176 (16.6)	4575 (21.5)	8751 (18.9)	1285 (16.7)	448 (16.5)	1733 (16.7)
15-17	18 807 (74.8)	10 895 (51.2)	29 702 (64.0)	5725 (74.5)	1749 (64.5)	7474 (71.9)
Sex						
Male	21 573 (85.8)	15 864 (74.6)	37 437 (80.6)	6581 (85.7)	2183 (80.5)	8764 (84.3)
Female	3582 (14.2)	5406 (25.4)	8988 (19.4)	1101 (14.3)	529 (19.5)	1630 (15.7)
Race/ethnicity						
White	3983 (16.3)	7977 (38.8)	11 960 (26.6)	1422 (19.0)	857 (32.7)	2279 (22.6)
African American	15 019 (61.3)	6397 (31.1)	21 416 (47.5)	4257 (56.9)	748 (28.6)	5005 (49.6)
Hispanic	4322 (17.6)	4643 (22.6)	8965 (19.9)	1444 (19.3)	777 (29.7)	2221 (22.0)
Other	1187 (4.8)	1527 (7.4)	2714 (6.0)	353 (4.7)	236 (9.0)	589 (5.8)
Insurance						
Private	5583 (27.9)	6473 (36.8)	12 056 (32.1)	1892 (30.8)	819 (37.2)	2711 (32.5)
Medicaid	10 775 (53.9)	8762 (49.7)	19 537 (52.0)	3487 (56.7)	1112 (50.6)	4599 (55.1)
Self-pay	3621 (18.1)	2380 (13.5)	6001 (16.0)	770 (12.5)	269 (12.2)	1039 (12.4)
Intent						
Assault	19 057 (79.9)	8543 (40.6)	27 600 (61.5)	5684 (77.9)	1715 (63.7)	7399 (74.1)
Self-inflicted	1189 (5.0)	1875 (8.9)	3064 (6.8)	607 (8.3)	227 (8.4)	834 (8.4)
Unintentional	3606 (15.1)	10 629 (50.5)	14 235 (31.7)	1005 (13.8)	750 (27.9)	1755 (17.6)

Firearm injuries resulted in a mean (SD) ISS of 10.9 (12.7) points compared with 4.6 (6.8) points for cut or pierce injuries. Mean (SD) hospital LOS was 5.0 (8.4) days for firearm injuries and 2.8 (4.1) days for cut or pierce injuries. In isolating the subcohort of children with critical injuries (ie, resulting in ICU admission), we identified 7682 firearm injuries (30.5%) and 2712 cut or pierce injuries that required ICU care (12.8%). Mean (SD) ISS for critical injuries was 17.2 (11.7) points for firearm injuries and 11.0 (10.3) points for cut or pierce injuries. Mean (SD) ICU LOS was 5.1 (7.7) days for critical firearm injuries and 3.1 (4.5) days for critical cut or pierce injuries. For similar numbers of firearm injuries and cut or pierce injuries over the study period, firearm injuries accounted for a total of 126 027 hospital days and 39 255 ICU days, whereas cut or pierce injuries accounted for 58 705 hospital days and 8353 ICU days. Of the critical injuries, 3409 (44.4%) of firearm injuries required mechanical ventilation, and 699 (25.8%) of cut or pierce injuries required mechanical ventilation (Table 2).

**Table 2.** Clinical Characteristics of All Injuries and Critical Injuries

Variable	Firearm Injury	Cut or Pierce Injury	Total
**All Injuries**			
ISS			
Mean (SD)	10.9 (12.7)	4.6 (6.8)	8.0 (10.9)
Median (IQR)	9 (1-16)	1 (1-5)	4 (1-10)
Hospital LOS, d			
Mean (SD)	5.0 (8.4)	2.8 (4.1)	4.0 (6.8)
Median (IQR)	2 (1-6)	1 (1-3)	2 (1-4)
ICU admission, frequency (%)	7682 (30.5)	2712 (12.8)	10394 (22.4)
Mortality, frequency (%)	1688 (6.7)	120 (0.6)	1808 (3.9)
**Critical Injuries**			
ISS			
Mean (SD)	17.2 (11.7)	11.0 (10.3)	15.6 (11.7)
Median (IQR)	16 (9-25)	9 (4-14)	13 (9-25)
Hospital LOS, d			
Mean (SD)	10.4 (12.4)	6.5 (7.7)	9.4 (11.5)
Median (IQR)	3 (1-5)	2 (1-3)	2 (1-5)
ICU LOS, d			
Mean (SD)	5.1 (7.7)	3.1 (4.5)	4.6 (7.0)
Median (IQR)	3 (1-5)	2 (1-3)	2 (1-5)
Ventilator frequency, %	3409 (44.4)	699 (25.8)	4108 (39.5)
Ventilator days, No.			
Mean (SD)	4.96 (7.70)^a^	3.2 (5.6)^a^	4.7 (7.4)^a^
Median (IQR)	2 (1-5)	2 (1-3)	2 (1-5)

Abbreviations: ICU, intensive care unit; IQR, interquartile range; ISS, Injury Severity Score (range: 0-75 points, with injuries scored <9 as minor, 9-15 as moderate, >15 as severe, and 75 as unsurvivable); LOS, length of stay.

^a^ *P* < .001.

**Figure 1.**
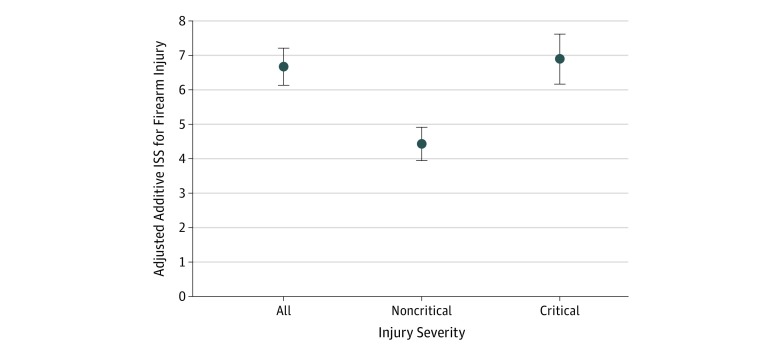
Adjusted Additive Higher Injury Severity Score (ISS) Associated With Firearm Compared With Cut or Pierce Injuries Compared with cut or pierce injuries, firearm injuries had a 6.7-point (95% CI, 6.1-7.2; *P* < .001) higher ISS for all injuries, a 6.9-point (95% CI, 6.2-7.6; *P* < .001) higher score for critical injuries, and a 4.4-point (95% CI, 3.9-4.9; *P* < .001) higher score for noncritical injuries. Error bars indicate 95% CI.

A multivariable logistic regression model adjusted for age, sex, data year, and clustering by facility demonstrated that those with firearm injuries had an RR of 2.3 (95% CI, 2.1-2.5; *P* < .001) for requiring ICU admission compared with those with cut or pierce injuries. Generalized linear regression adjusting for age, sex, data year, and clustering by facility demonstrated that firearm injuries compared with cut or pierce injuries were associated with a 6.7-point (95% CI, 6.1-7.2) higher ISS for all injuries, 6.9-point (95% CI, 6.2-7.6) higher ISS for critical injuries, and a 4.4-point (95% CI, 3.9-4.9) higher ISS for noncritical injuries (*P* < .001) (Figure 1).

**Figure 2.**
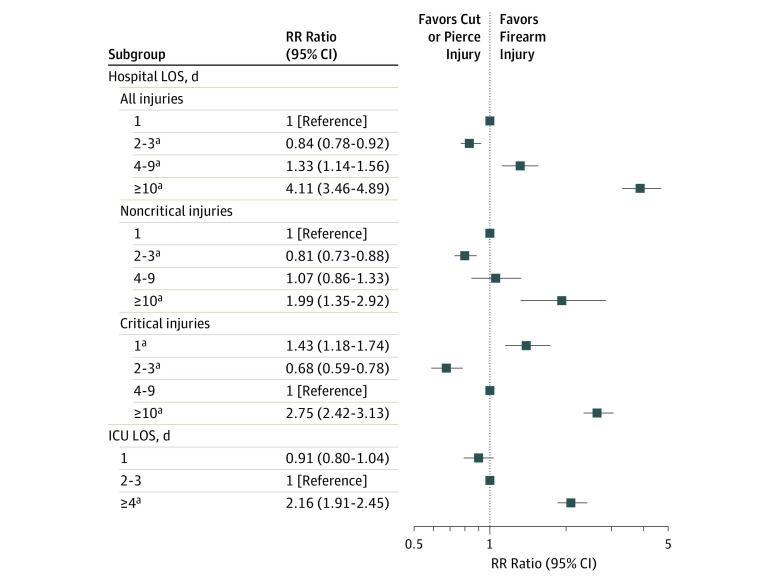
Hospital and Intensive Care Unit (ICU) Length of Stay (LOS) Relative Risk (RR) Ratios for Firearm Compared With Cut or Pierce Injuries ^a^*P* < .001.

Multinomial logistic regression adjusted for age, sex, data year, and clustering by facility revealed distinct patterns for LOS. Those with firearm injuries were less likely to have hospital LOS of 2 to 3 days (RR ratio, 0.84; 95% CI, 0.78-0.92; *P* < .001) and were more likely to have hospital LOS of 10 or more days (RR ratio, 4.11; 95% CI, 3.46-4.89; *P* < .001) compared with those with cut or pierce injuries and with 1-day hospital LOS. Those with critical firearm injuries were more likely to have ICU LOS of 4 or more days (RR ratio, 2.16; 95% CI, 1.91-2.45; *P* < .001) compared with cut or pierce injuries and with 2- to 3-day ICU LOS (Figure 2).

The sensitivity analysis revealed similar estimates to primary analysis and resulted in similar conclusions for all outcomes. Those with firearm injuries had an RR of 2.3 (95% CI, 2.1-2.5; excluded ISS, 75 points) or 2.2 (95% CI, 1.9-2.3; excluded self-inflicted injuries) compared with those with cut or pierce injuries to require ICU admission (main analysis RR, 2.3; 95% CI, 2.1-2.5). When excluding injuries with an ISS of 75 points or self-inflicted injuries, firearm injuries were also associated with a higher ISS in all injury subcohorts compared with cut or pierce injuries (excluding 75-point ISS: 5.7-point [95% CI, 5.3-6.1] higher ISS for all injuries, 3.3-point [95% CI, 3.0-3.6] higher for noncritical injuries, and 6.8-point [95% CI, 6.2-7.4] higher for critical injuries; excluding self-inflicted injuries: 6.0-point [95% CI, 5.6-6.4] higher ISS for all injuries, 4.1-point [95% CI, 3.6-4.5] higher for noncritical injuries, and 5.8-point [95% CI, 5.1-6.5] higher for critical injuries).

Similar patterns were found in hospital LOS and ICU LOS. Those with cut or pierce injuries were more likely to have short (2-3 days) hospital LOS (excluding 75-point ISS: RR ratio, 0.86 [95% CI, 0.79-0.94]; excluding self-inflicted injuries: RR ratio, 0.88 [95% CI, 0.81-0.96]) and short (1 day) ICU LOS (excluding 75-point ISS: RR ratio, 0.90 [95% CI, 0.79-1.03]; excluding self-inflicted injuries: RR ratio, 0.89 [95% CI, 0.78-1.02]). Those with firearm injuries were more likely to have prolonged (>10 days) hospital LOS (excluding 75-point ISS: RR ratio, 4.22 [95% CI, 3.54-5.02]; excluding self-inflicted injuries: RR ratio, 4.60 [95% CI, 4.11-5.16]) and prolonged (>4 days) ICU LOS (excluding 75-point ISS: RR ratio, 2.18 [95% CI, 1.92-2.46]; excluding self-inflicted injuries: RR ratio, 2.16 [95% CI, 1.89-2.47]) (eTable 1 and eTable 2 in the Supplement).

Sensitivity analysis for LOS demonstrated similar results as the primary analysis. Firearm injuries were associated with more days of hospitalization compared with cut or pierce injuries for all injuries (incidence rate ratio [IRR], 1.77; 95% CI, 1.66-1.89) and noncritical firearm injuries (IRR, 1.16; 95% CI, 1.06-1.28). Critical firearm injuries were associated with more days of hospitalization (IRR, 1.61; 95% CI, 1.52-1.71) and more days of ICU stay (IRR, 1.69; 95% CI, 1.57-1.82) compared with critical cut or pierce injuries (eTable 3 in the Supplement).

## Discussion

Limited information is available to date on short-term clinical outcomes and resource utilization in pediatric patients who sustain a firearm injury. This study expands that evidence and, to our knowledge, is one of the first to contextualize pediatric firearm injuries in the broader scope of penetrating sharp force injuries at the national level, especially among those with critical injury. These findings indicate a notable distinction for firearm injuries. In addition to the previously known high case fatality, this study found that a high burden of injury severity and resource utilization was associated with the use of firearms, including among the cohort of children with critical injury.^10,14,19^

In our comparison, firearm injuries were associated with an almost 7-point higher mean ISS compared with cut or pierce injuries. The mean (SD) ISS for firearm injuries was greater than twice the ISS for cut or pierce injuries (10.9 [12.7] points vs 4.6 [6.8] points). Among critical injuries, the mean (SD) ISS was more than 50% greater for firearm injuries compared with cut or pierce injuries (17.2 [11.7] points vs 11.0 [10.3] points). These additional severity points associated with firearm injury reflect a clinical distinction between firearm injuries and cut or pierce injuries. The mean ISS score for cut or pierce injuries is categorized as mild (<9 points) compared with moderate for firearm injuries (9-15 points). In critical injuries, the mean ISS score for cut or pierce injuries is categorized as moderate compared with severe for firearm injuries (>15 points). This higher injury severity category associated with the additional ISS points for firearm injury represents an increased risk of morbidity and mortality. This distinction is maintained even when examining in isolation the cohort of children with critical injury, which suggests a clinical distinction between firearm injuries and cut or pierce injuries across the spectrum of injury severity.

In addition, we demonstrated that, compared with children with a cut or pierce injury, those with a firearm injury were more than twice as likely to be critically injured and require ICU admission. Children injured by firearms were more likely to have a longer hospital LOS. Despite similar numbers of patient encounters for firearm injuries and cut or pierce injuries, firearm injuries resulted in nearly 2-fold greater hospital days and nearly 5-fold greater ICU days. These markers of short-term and long-term outcomes are important because ICU admission and longer LOS are associated with increased risk for morbidity and complications and reflect a high economic burden and high resource utilization.

High-velocity and impact bullets carry considerable force that is higher than that of many other tools that can inflict a cut or pierce injury. When the bullet transfers force to a human body on impact, it causes substantial damage to local and surrounding structures. A bullet, for example, can easily cause injuries to structures such as the brain, whereas a knife would be unable to penetrate the skull. Similarly, bullets can be fired in rapid succession, and therefore a child can sustain multiple injuries to different areas of the body. The potential for greater local damage and more injury locations increases the likelihood of a firearm injury to a vital area, such as a vascular structure, a nerve, or hollow viscous area that requires a surgical intervention. We did not examine the number of procedures performed, but this number could also explain the longer ICU and hospital LOS in firearm injuries if patients required more operative interventions as a result of their injury. Given that ISS scoring takes into account various injury locations and generates a composite score, injury to multiple areas of the body could account for a higher ISS in children injured by firearms compared with children injured by nonfirearm penetrating trauma mechanisms.

We considered 2 additional higher-severity markers associated with firearm injuries: higher mortality in firearm injuries and self-inflicted firearm injuries, which often take the form of gunshot wounds to the head. We found no meaningful differences in the primary conclusions when excluding patients who had self-inflicted injuries or patients with an ISS equal to 75 (an unsurvivable injury). Firearm injuries were still associated with a higher ISS, higher likelihood of ICU admission, and higher likelihood of long hospital LOS and ICU LOS.

These findings suggest that injury severity in firearm injuries, compared with cut or pierce injuries, is not driven solely by fatalities or self-inflicted injuries. These findings also strengthen the conclusions about the distinct injury burden inflicted by firearms.

Previous studies have documented the ISS and ICU admission percentage in children with a firearm injury.^5,6,8,13,16,17,20,26,29^ Comparing ICU percentages and ISSs between studies is not a straightforward task, because different databases capture varying cohorts of children, and several previous studies were single-center studies. We believe this study adds to the understanding of the distinctiveness of firearm injury among a larger group of pediatric trauma injuries by comparing its aspects with those of cut or pierce injury.

### Limitations

This study has several limitations. First, although the NTDB contains the largest collection of trauma data, its population of firearm injuries and cut or pierce injuries is not fully comprehensive and does not capture every pediatric firearm and cut or pierce encounter over the 10-year study period. Injuries that result in death at the scene are not included in the NTDB. Such deaths are more likely to occur in firearm injuries and result in a subset of fatal firearm injuries, which were excluded from this study. Second, the NTDB is a collection of encounter-level data. As a result, linking encounters by patient or identifying which patients had repeated injuries during the study period was not possible. To address this issue, we eliminated all encounters coded as *transferred* to avoid the double counting of injuries. This data omission ultimately likely removed a subset of pediatric patients who were critically injured and transferred to a hospital with expertise in pediatric trauma, as data were then only available from these patients’ initial hospitalization. Lack of this information likely affected firearm injuries and cut or pierce injuries similarly and may have resulted in a small decrease in the sample size and power of this study. Other limitations inherent in the use of the NTDB were the lack of granular descriptions of injuries, longitudinal follow-up data, and cost or monetary measures, which were not collected.

Future research should focus on possible explanations for the increased severity of pediatric firearm injuries compared with cut or pierce injuries; examine data on procedures, complications, and disposition for injury encounters; and address ongoing morbidity from these injuries. In addition, further studies should use the most recent years of data, when available, to capture the changing epidemiological profile of pediatric firearm injuries, especially the recent increases in suicide attempts via firearms.

## Conclusions

This study found greater injury severity and resource utilization associated with pediatric firearm injuries compared with other penetrating sharp force injuries, including among critical trauma. This finding raises the important consideration of means. Firearms pose a notably high morbidity risk. Given these findings, we believe that reducing pediatric firearm injuries through legislative efforts, safe storage practices, and community-based interventions is vital to the safety of children in the United States.

## Notes

Supplement. **eTable 1. **Summary of Results for Sensitivity Analysis Excluding Encounters With ISS=75 **eTable 2.** Summary of Results for Sensitivity Analysis Excluding Encounters for Self-Inflicted Injuries **eTable 3.** Sensitivity Analysis Using Poisson Regression for Hospital and ICU LOS
