# Supplementary material for: Evaluation of Injury Severity and Resource Utilization in Pediatric Firearm and Sharp Force Injuries
Source: JAMA Netw Open. 2019 Oct 9;2(10):e1912850. doi: 10.1001/jamanetworkopen.2019.12850 (PMC6802232; doi:10.1001/jamanetworkopen.2019.12850)
Supplement: Supplement. — eTable 1. Summary of Results for Sensitivity Analysis Excluding Encounters With ISS=75 eTable 2. Summary of Results for Sensitivity Analysis Excluding Encounters for Self-Inflicted Injuries eTable 3. Sensitivity Analysis Using Poisson Regression for Hospital and ICU LOS [file jamanetwopen-2-e1912850-s001.pdf]

Supplementary Online Content

Wolf AE, Garrison MM, Mills B, Chan T, Rowhani-Rahbar A. Evaluation of injury severity and resource utilization in pediatric firearm and sharp force injuries. *JAMA Netw Open*. 2019;2(10):e1912850. doi:10.1001/jamanetworkopen.2019.12850

eTable 1. Summary of Results for Sensitivity Analysis Excluding Encounters With ISS=75

eTable 2. Summary of Results for Sensitivity Analysis Excluding Encounters for Self-Inflicted Injuries

eTable 3. Sensitivity Analysis Using Poisson Regression for Hospital and ICU LOS

This supplementary material has been provided by the authors to give readers additional information about their work.

© 2019 Wolf AE et al. *JAMA Network Open*

eTable 1. Summary of Results for Sensitivity Analysis Excluding Encounters With ISS=75

Outcome	Estimate (95%CI)	p-value
**ICU Admission**	**RR** Firearm Injuries Compared to Cut/Pierce Injuries: 2.30 (2.07-2.54)	<0.001
**ISS**	**Additive Points** for Firearm Injuries Compared to Cut/Pierce Injuries	
All Injuries	5.69 (5.26-6.13)	<0.001
Non-Critical Injuries	3.29 (2.95-3.63)	<0.001
Critical Injuries	6.78 (6.15-7.40)	<0.001
**Hospital LOS**	**RRR** For Firearm Compared to Cut/Pierce Injury	
All Injuries		
1 Day	Reference	
2-3 Days	0.86 (0.79-0.94)	<0.01
4-9 Days	1.37 (1.17-1.61)	<0.001
10+ Days	4.22 (3.54-5.03)	<0.001
Non-Critical Injuries		
1 Day	Reference	
2-3 Days	0.83 (0.75-0.91)	<0.001
4-9 Days	1.10 (0.88-1.37)	0.41
10+ Days	2.04 (1.38-3.00)	<0.001
Critical Injuries		
1 Days	1.38 (1.14-1.68)	<0.01
2-3 Days	0.67 (0.58-0.76)	<0.001
4-9 Days	Reference	
10+ Days	2.74 (2.40-3.12)	<0.001
**ICU LOS**	**RRR** For Firearm Compared to Cut/Pierce Injury	
1 Day	0.90 (0.79-1.03)	0.11
2-3 Days	Reference	
4+ Days	2.18 (1.92-2.46.)	<0.001

All models adjusted for age, sex, year, and clustering by facility
Abreviations: ICU, intensive care unit; ISS, injury severity score; LOS, length of stay; RR, relative risk; RRR, relative risk ratio

© 2019 Wolf AE et al. *JAMA Network Open*

eTable 2. Summary of Results for Sensitivity Analysis Excluding Encounters for Self-Inflicted Injuries

Outcome	Estimate (95%CI)	p-value
**ICU Admission**	**RR** Firearm Injuries Compared to Cut/Pierce Injuries: 2.19 (2.01-2.39)	<0.001
**ISS**	**Additive Points** for Firearm Injuries Compared to Cut/Pierce Injuries	
All Injuries	6.01 (5.58-6.44)	<0.001
Non-Critical Injuries	4.07 (3.62-4.52)	<0.001
Critical Injuries	5.75 (5.05-6.46)	<0.001
**Hospital LOS**	**RRR** For Firearm Compared to Cut/Pierce Injury	
All Injuries		
1 Day	Reference	
2-3 Days	0.88 (0.81- 0.96)	<0.01
4-9 Days	1.50 (1.38-1.64)	<0.001
10+ Days	4.60 (4.11- 5.16)	<0.001
Non-Critical Injuries		
1 Day	Reference	
2-3 Days	0.84 (0.77-0.92)	<0.001
4-9 Days	1.25 (1.13-1.38)	<0.001
10+ Days	2.62 (2.23- 3.07)	<0.001
Critical Injuries		
1 Days	1.22 (1.00-1.50)	0.05
2-3 Days	0.67 (0.58-0.77)	<0.001
4-9 Days	Reference	
10+ Days	2.71 (2.37-3.10)	<0.001
**ICU LOS**	**RRR** For Firearm Compared to Cut/Pierce Injury	
1 Day	0.89 (0.78-1.02)	0.10
2-3 Days	Reference	
4+ Days	2.16 (1.89- 2.47)	<0.001

All models adjusted for age, sex, year, and clustering by facility
Abreviations: ICU, intensive care unit; ISS, injury severity score; LOS, length of stay; RR, relative risk; RRR, relative risk ratio

© 2019 Wolf AE et al. *JAMA Network Open*

eTable 3. Sensitivity Analysis Using Poisson Regression for Hospital and ICU LOS

**Outcome**	**IRR (95%CI) For Firearm Compared to Cut/Pierce Injury**	**p-value**
**Hospital LOS**		
All Injuries	1.77 (1.66-1.89)	<0.001
Non-Critical Injuries	1.16 (1.06-1.28)	<0.01
Critical Injuries	1.61 (1.52-1.71)	<0.001
**ICU LOS**	1.69 (1.57-1.82)	<0.001

Model adjusted for age, sex, year, and clustering by facility
Abreviations: ICU, intensive care unit; IRR, incidence rate ratio; LOS, length of stay

© 2019 Wolf AE et al. *JAMA Network Open*
